# Measuring shared decision making in oncology: Development and first testing of the iSHAREpatient and iSHAREphysician questionnaires

**DOI:** 10.1111/hex.13015

**Published:** 2020-02-05

**Authors:** Hanna Bomhof‐Roordink, Fania R. Gärtner, Nanny van Duijn‐Bakker, Trudy van der Weijden, Anne M. Stiggelbout, Arwen H. Pieterse

**Affiliations:** ^1^ Medical Decision Making Department of Biomedical Data Sciences Leiden University Medical Center Leiden The Netherlands; ^2^ Department of Family Medicine CAPHRI School for Care and Public Health Research Institute Maastricht University Maastricht The Netherlands

**Keywords:** content validity, field‐testing, formative, oncology, questionnaire, shared decision making

## Abstract

**Background:**

Existing measures to assess shared decision making (SDM) have often been developed based on an ill‐defined underlying construct, and many assess physician behaviours only or focus on a single patient‐physician encounter.

**Objective:**

To (a) develop a patient and a physician questionnaire to measure SDM in oncology and (b) determine their content validity and comprehensibility.

**Methods:**

A systematic review of SDM models and an oncology‐specific SDM model informed the domains of the SDM construct. We formulated items for each SDM domain. Cancer patients and physicians rated content validity in an online questionnaire. We assumed a formative measurement model and performed online field‐testing in cancer patients to inform further item reduction. We tested item comprehension in cognitive interviews with cancer patients and physicians.

**Results:**

We identified 17 domains and formulated 132 items. Twelve cancer patients rated content validity at item level, and 11 physicians rated content validity at domain level. We field‐tested the items among 131 cancer patients and conducted cognitive interviews with eight patients and five physicians. These phases resulted in the 15‐item iSHAREpatient and 15‐item iSHAREphysician questionnaires, covering 13 domains.

**Conclusions:**

We thoroughly developed the iSHARE questionnaires. They both assess patient and physician behaviours and cover the entire SDM process rather than a single consultation.

## 1 BACKGROUND

Developing a measurement instrument is not something to be done on a rainy Sunday afternoon. If it is done properly, it may take years.

de Vet et al – ‘Measurement in Medicine’^1^

Shared decision making (SDM) between patient and physician is considered the pinnacle of patient‐centred care.^2^ As a consequence, there is an urge to establish existing SDM levels and to detect the effect of SDM training and interventions. Measurement instruments to assess the SDM process exist but have demonstrated several issues relating to what they intend to assess and how they have been developed. Recent systematic reviews of SDM measurement instruments concluded that developers often do not or only vaguely define the underlying construct,^3^ and that available SDM measurement instruments substantially differ in the domains that they cover.^4^ Patient behaviour is part of SDM models,^5^but often‐used SDM measurement instruments only assess physician behaviour (eg OPTION,^6^ CollaboRATE^7^) or include physician behaviour when assessing patient's weighing of treatment options (eg SDM‐Q‐9,^8^ SDM‐Q‐Doc^9^), impeding a transparent assessment of patient's role. The scope of SDM assessments is usually limited to a single consultation, while SDM extends to time outside consultations and is not confined to the space where the patient and physician meet.^10^, ^11^ There is growing awareness of the need for a valid measurement instrument that is capable of capturing the entire SDM process. Such a measurement instrument should be based on a clearly defined construct and include both patient and physician behaviours, during as well as outside consultations.

Existing SDM measurement instruments vary in terms of the viewpoint from which SDM is reported. This can either be that of an independent observer (eg OPTION‐5^12^), the patient (eg SDM‐Q‐9,^8^ CollaboRATE^7^), the physician (eg SDM‐Q‐Doc^9^) or a combination thereof (eg MAPPIN’SDM^13^). Overall, agreement between the different viewpoints has been found to be poor.^14^, ^15^, ^16^, ^17^, ^18^ Recently again, a poor agreement (*r* = .14) between the SDM‐Q‐9 and SDM‐Q‐doc was found in an oncology setting.^19^ Possibly, discrepancies occur because patients and physicians have different perspectives on what SDM entails and because they seldom have been involved in the development of SDM measurement instruments to date. Moreover, guidelines on the evaluation of psychometric properties of health measurement instruments recommend that the target group (ie researchers, patients and/or physicians) should be involved in content validity testing,^1^ next to conducting cognitive interviews. This has occurred for only six of the 40 existing SDM measurement instruments.^3^

We set out to develop a questionnaire based on an explicit underlying construct, and observing further recommendations on the development of measurement instruments.^1^ We considered a questionnaire most appropriate to develop as recording and coding consultations is a time‐consuming process. Further, we posit that for the assessment of SDM a formative measurement model should be assumed.^3^, ^20^, ^21^, ^22^ That is, we view the SDM process as a composite construct that is the result of independent indicators (ie the items *form* the construct), which can, but need not, be correlated with each other. In contrast, the developers of most available SDM measurement instruments have assumed a reflective measurement model,^3^ in which the latent SDM construct is responsible for the scores on the indicators (ie the items *reflect* the construct).^1^

We decided to develop an SDM questionnaire for the oncology setting because cancer patients often face preference‐sensitive decisions^23^, ^24^ that call for SDM.^25^ Cancer patients likely feel highly vulnerable,^26^ and decisions need to be made about treatment options that often have severe and irreversible side‐effects. At the same time, high levels of uncertainty may exist,^24^ and time is often a constraint.^27^ We further preferred an oncology‐specific questionnaire, as definitions of SDM differ between health‐care settings.^5^

Therefore, the present study aimed to (a) develop a patient and a physician questionnaire to measure SDM in oncology and (b) determine their content validity and comprehensibility.

## 2 METHODS

### 2.1 Study design

We aimed to develop short questionnaires to measure SDM from the patient and the physician viewpoint, with the same items formulated from the two different perspectives. We even preferred the physician questionnaire to contain a smaller number of items, all part of the patient questionnaire.

**Figure 1:**
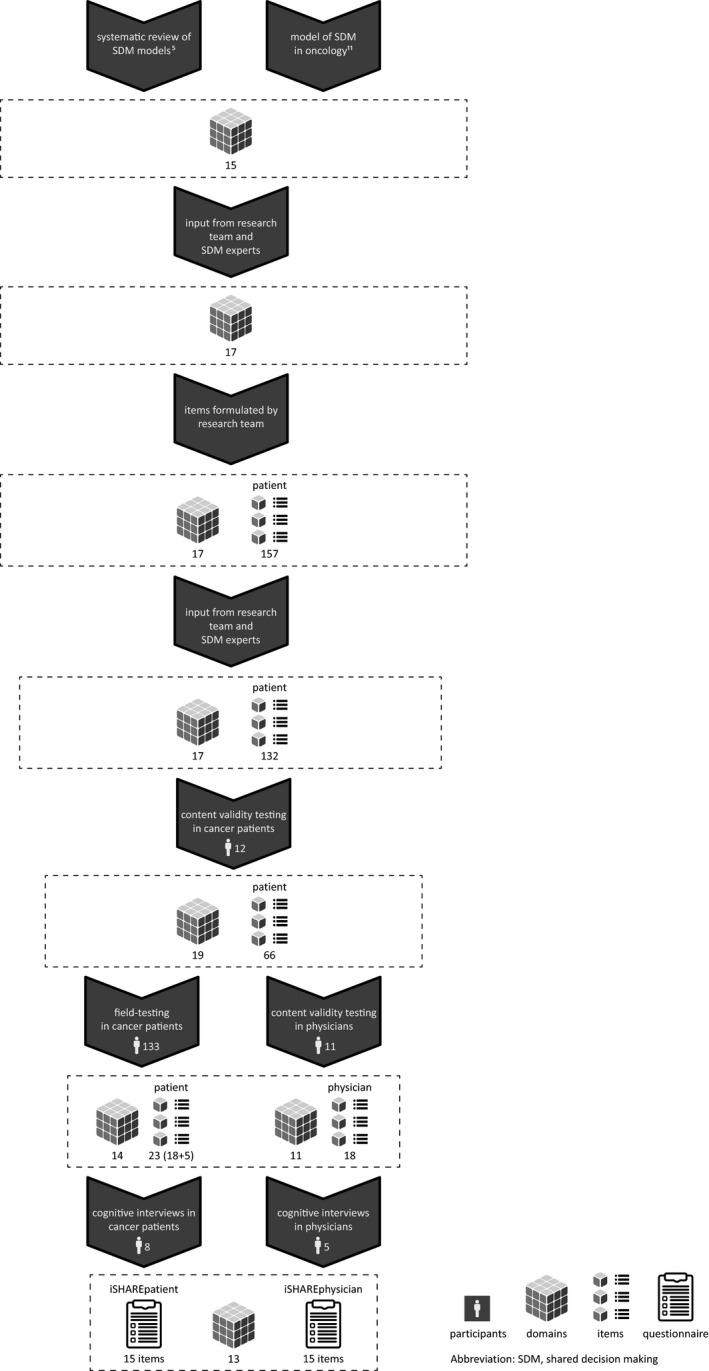
Visual representation of the development process of the iSHARE questionnaires

We used the COnsensus‐based Standards for the selection of health Measurement Instruments (COSMIN) checklist as a guideline throughout the development process.^28^ We describe the different phases in more detail in the sections below and in Figure 1. In sum, we selected domains to define the SDM construct; created an item pool to assess the domains; tested content validity (ie relevance and comprehensiveness) of the item pool in cancer patients and of the domains in physicians and performed a field‐test to further inform the selection of domains and items; and determined comprehensibility of the draft versions of the questionnaires in in‐person cognitive interviews. Note that the selection of items was informed by the results obtained by field‐testing and not based on internal consistency testing and factor analysis, since we assumed a formative measurement model.^1^ Further, throughout the development process our goal was to assess domains that were essential for SDM in oncology, in order to be *specific* rather than comprehensive. We adopted this approach so that we would include domains that were unique to SDM and would assess *shared* decision making rather than other decision‐making models. Also, we focused on observable behaviour, assuming that this will contribute to achieving more agreement between patients’ and physicians’ viewpoints when assessing SDM. We performed a side‐study to determine the most appropriate and feasible response scale for the questionnaires and tested several formats during the cognitive interviews (see Section 2.7), to select the final response scale.^29^

### 2.2 Participant recruitment

For content validity testing in patients, we approached cancer patients aged ≥18 years and able to speak and write Dutch, via their physician at the LUMC, through either a letter or during a consultation. Patients willing to participate sent their written informed consent to the researcher and then received the link to the online survey. For field‐testing, we approached cancer patients participating in an online panel (Kanker.nl), who had agreed to be approached for research, by e‐mail and sent them the link to the online survey. They provided informed consent by checking a box at the start of the survey. For the cognitive interviews, we approached cancer patients as described for content validity testing and scheduled an interview at the LUMC. They received reimbursement for travel expenses. We asked for patients’ age and education. The patients further reported their diagnosis (field‐testing) or it was obtained from their treating physician (content validity testing and cognitive interviews).

For content validity testing in physicians, we approached physicians treating cancer patients from one Dutch academic hospital (LUMC) and from two Dutch non‐academic hospitals (Haga Hospital, The Hague, and Reinier de Graaf, Delft) by e‐mail and sent them the link to the online survey. For the cognitive interviews, we approached physicians from the LUMC by e‐mail and if they agreed to participate, we scheduled an interview at their workplace.

### 2.3 Construct definition and item pool creation

To determine the SDM construct, we made a first selection of domains based on (a) an SDM model in oncology informed by the views of cancer patients, health‐care professionals and SDM researchers,^11^ and (b) the first search (up to June 21, 2016) for a systematic review of SDM models across settings.^5^

Next, we shared the list of domains with international SDM experts and discussed it first by e‐mail and then in person at the 2017 International Shared Decision Making Conference in Lyon, France. The research team made a definitive selection of domains forming the SDM construct.

Finally, we created an item pool for the patient questionnaire by formulating five or more potential items per domain. If available, we used phrasings that patients had used in an earlier interview study^11^ and included relevant items from the SDM‐Q‐9.^30^ We asked the international SDM experts for feedback on how well the proposed items reflected the domains, and the research team made a definitive selection of items to present to patients during content validity testing.

### 2.4 Content validity testing in patients

First, we pilot‐tested questions asking to rate the importance of each item for the domain to which it belonged among two research assistants from outside the research team. As they both considered almost all items to be very important, we decided it to be more informative to ask patients to select the most important items for each domain. Specifically, we presented the patients with the name and description of each domain of SDM in oncology together with the proposed items and asked them to choose the three items that they considered most important for each domain. We further asked them to indicate per domain if the proposed items comprehensively represented it. We then presented the complete list of domains, without items, and asked the patients to indicate if they missed one or more domains, or considered one or more domains to be redundant. In the final step, we asked the patients to judge the clarity (yes/no) of the draft introduction of the iSHAREpatient questionnaire.

We aimed to narrow down the total number of selected items to approximately 50. Two researchers (NDB and HBR) independently selected the items to be used for assessing content validity in physicians and field‐testing in patients based on the results, discussed their selection and reached agreement in consultation with the research team.

### 2.5 Content validity testing in physicians

We asked physicians to rate the importance of each domain for SDM in oncology, described as ‘doctors and patients making decisions together about cancer treatment’, on a seven‐point scale ranging from ‘not important at all’ to ‘very important’. Next, we presented the complete list of domains and asked the physicians to indicate if they missed one or more domains or considered one or more domains to be redundant. We then asked which three domains describing patient behaviour and which six domains describing physician behaviour they considered most important for SDM in oncology*.* These numbers differed because the construct included more domains describing physician than patient behaviour. Finally, in order to create a physician questionnaire that would be as short as possible, we asked which four to six domains of the complete list they considered indispensable in order to assess SDM with a physician questionnaire.

### 2.6 Field‐testing in patients

We asked patients to rate the importance of each item for each domain, on a seven‐point scale ranging from ‘not important at all’ to ‘very important’; to choose the most important item for each domain; and to indicate for each domain if they missed one or more items. We then presented the complete list of domains, without items, and asked the patients to indicate if they missed one or more domains or considered one or more domains to be redundant. We finally asked which three domains describing patient behaviour and which six domains describing physician behaviour they considered most important for SDM in oncology.

We selected domains for the draft patient questionnaire informed by the results from the field‐testing in patients and the content validity testing in physicians. We selected items for the draft patient questionnaire informed by the results from the field‐testing in patients. We selected domains for the draft physician questionnaire informed by the four to six domains chosen by physicians in the final step of content validity testing. The items for the draft physician questionnaire were taken from the draft patient questionnaire, but formulated from the physician's viewpoint.

### 2.7 Cognitive interviews in patients and physicians

Two trained researchers (NDB, HBR) conducted individual interviews with patients using the draft patient questionnaire and with physicians using the draft physician questionnaire. We determined comprehensibility of the introduction, the items and several response scales, and we assessed if items should be removed, replaced or adapted. We adapted the draft questionnaires between interviews, based on the responses. Finally, we made the decision to align the two questionnaires for sake of comparability and, to that end, selected the same items covering the same domains in the two questionnaires, formulated from the different viewpoints.

## 3 RESULTS

### 3.1 Sample characteristics

For the formulation of the construct, we approached five international SDM experts to give feedback on our initial selection of domains, of which four responded and two also participated in the in‐person meeting. For the feedback on the items, the same five international SDM experts were approached and three of them responded.

In total, 153 patients and 16 physicians participated in this study (Table 1). For content validity testing, 14 patients initially provided informed consent and 12 of them completed the survey. Eleven of the 18 physicians who we approached participated. In total, 185 patients started with the field‐test survey, and 133 completed it. Non‐completers (N = 52) did not significantly differ from completers regarding age, level of education or gender. Ten patients provided informed consent to participate in the cognitive interviews of whom eight were interviewed. Five of the six physicians who we approached participated in the cognitive interviews.

**Table 1:** Characteristics of the participants by study phase

	N or mean (SD)	N or mean (SD)	N or mean (SD)
	Content validity testing	Field‐testing	Cognitive interviews
Cancer patients	12	133	8
Sex, female	7	75	7
Age, y	67.8 (8.9)	58.9 (10.8)	63.0 (11.6)
Primary tumour type^a^			
Breast	0	30	2
Urological	4	25	1
Haematological	0	21	0
Gastrointestinal	0	20	4
Otolaryngology	0	9	0
Gynaecological	5	7	0
Lung	3	7	1
Skin	0	5	0
Other	0	9	0
Treatment intent			
Curative	8		5
Palliative	4		3
Education level			
Low	2	8	2
Intermediate	4	52	0
High	6	73	6
Physicians	11		5
Sex, female	4		1
Age, y	51.9 (7.7)		48.8 (9.1)
Years since the start of specialist training	20.2 (8.2)		18.8 (8.5)
Specialty			
Surgery	3		1
Gynaecology	2		1
Pulmonology	2		0
Radiotherapy	2		1
Medical oncology	1		1
Urology	1		1

a Patients participating in the field‐testing could indicate more than one cancer diagnosis; 10 patients reported >1 diagnosis.

### 3.2 Construct definition and item pool creation

The integration of the findings from the SDM model in oncology and the systematic review resulted in a first selection of 15 domains to define the construct of SDM in oncology (Appendix 1). We clustered the domains by content in six dimensions. We added two domains informed by feedback from the SDM experts. The 17 domains related to both patient and physician behaviours. We then formulated five to 16 items per domain, resulting in a total list of 157 items to start with. Some items were then removed, reformulated or added based on feedback from the SDM experts, resulting in five to 11 items per domain, adding up to 132 items.

### 3.3 Content validity testing in patients

We presented the 17 domains with the 132 corresponding items to 12 patients. A number of items that the patients often selected in their top three across domains represented a separate domain, that is ‘The physician offers room for the patient to contribute to SDM’, which was added. Further, it was decided to split the domain ‘The patient considers what is most important to him/her in the context of the treatment options’ into a variant *inside* versus *outside* the consultation. Content validity testing in patients thus resulted in the selection of 19 domains and 66 corresponding items. Eleven of the 12 patients considered the introduction to be clear.

### 3.4 Content validity testing in physicians and field‐testing in patients

Eleven physicians assessed content validity of the 19 domains, and during field‐testing 133 patients rated the importance of 66 items considering the 19 domains. The respective selection processes resulted in 14 domains with 23 corresponding items for the draft patient questionnaire, and in 11 domains with 18 corresponding items for the draft physician questionnaire. The 11 domains and corresponding items selected for the physician questionnaire were also part of the patient questionnaire.

### 3.5 Cognitive interviews in patients and physicians

Input to the patient and physician cognitive interviews were a draft 24‐item patient questionnaire and a draft 18‐item physician questionnaire, respectively. The introduction to both the patient and the physician questionnaire explicitly included a statement that the time that the patient and the physician spoke about the treatment options may have entailed one or more conversations. We removed the domain ‘Physician mentions treatment options’ and items that participants considered too much alike. We reworded items that were considered unclear**.**

The patients indicated that certain questions seemed very similar to each other, although they were asking about different domains. We therefore added a comment to the introduction to the patient questionnaire about the apparent similarity of questions. At the end of the introduction we added a question asking whether the patient considered the introduction to be clear, with the sole aim to stimulate them to actually read the introduction; there is no intent to actually use patients’ response to the item in the definitive questionnaire. Finally, we added a sentence to the introduction to the patient questionnaire stressing that the questionnaire is *not* about satisfaction with the physician.

### 3.6 The iSHAREpatient and iSHAREphysician questionnaires

We named the final versions of the questionnaires the iSHAREpatient (Box 2) and the iSHAREphysician (Box 3) questionnaire. They comprise the same construct, consisting of 13 domains, clustered in six dimensions (Box 1). These are assessed using the same 15 items, formulated from the two different viewpoints. Three items explicitly assess patient behaviour. Each item is scored on a six‐point scale that ranges from ‘not at all’ (0) to ‘completely’ (5). The questionnaires include two versions of the last item, depending on whether a decision has already been made or not, in order for the questionnaires to be suitable both before and after the final treatment decision has been made.

Box 1 **The construct of SDM in oncology; final selection of domains and corresponding items, and clustering of the 13 domains by dimension**

**Table:** 

**Dimension I: Choice awareness**
1. The physician establishes (creates or checks) choice awareness—*item 8*
The physician makes explicit or checks that patient knows that there is a choice to be made as there is more than one reasonable treatment option available for the condition
2. The physician expresses that patient opinion is important in process—*item 9*
The physician makes explicit that the patient's opinion about the treatment options and/or what the patient considers important matters, in making the decision about the most appropriate treatment strategy
**Dimension II: Medical information**
3. The physician provides information on the benefits/risks of the treatment options—*item 1, 2 and 6*
The physician explicitly identifies at least one possible benefit and one possible harm of each treatment option. The physician clarifies the trade‐off
4. The physician provides balanced information—*item 3*
The physician gives information in an objective, balanced, neutral way about each treatment option and its benefit(s) and harm(s)
5. The physician checks patient's understanding—*item 4 and 5*
The physician checks patient's understanding of the treatment options and their risks and benefits
6. The patient asks for clarification—*item 7*
The patient asks for clarification, if something about the treatment options is not clear to him/her and/or asks for more information
**Dimension III: Preferences**
7. The physician checks own understanding of patient's values, goals of care, concerns and/or preferences in the context of the treatment options—*item 10*
The physician makes sure to understand patient's values, goals of care, concerns and/or preferences either by explicitly asking clarifying questions or by summarizing what the patient told
8. The patient expresses values, feelings, concerns, thoughts and preferences in the context of the treatment options*—item 13*
The patient expresses feelings, thoughts, values, concerns and preferences openly. Either at the patient's or the physician's initiative
**Dimension IV: Deliberation**
9. The physician supports the patient in deliberation—*item 11*
The physician supports the patient in considering what is important to the patient in life in the context of his/her disease and the treatment options, for example by probing values and/or their rank order, and/or structuring and/or summarizing the thoughts expressed by the patient
10. The patient considers what is most important to him/her in the context of treatment options—*item 14*
The patient considers the treatment options based on what he/she has learned about them. He/she considers what is important to him/her in life in the context of his/her disease and the treatment options. He/she thinks about what he/she would want to achieve and would want to avoid. This may happen during as well as outside the consultation
**Dimension V: Time for deliberation**
11. The physician gives the patient room to contribute to SDM—*item 12*
The physician gives the patient room to contribute to SDM, by giving time and space for asking questions and/or expressing values, feelings, concerns, thoughts and preferences and/or considering the treatment options
**Dimension VI: Decision**
12. Make or explicitly postpone decision that is based on patient's preferences/values/goals—*item 15*
A treatment decision is explicitly made, based on patient's preferences/values/goals, either at the patient's or the physician's initiative
13. The physician assesses what the patient needs to make a decision—*item 16*
If the decision is postponed, the physician more or less explicitly ascertains what the patient needs in order to be able to determine what is important to him/her and/or determine his/her preferred option and/or make the decision, by himself/herself or together with the physician

Box 2 **iSHAREpatient^†^**

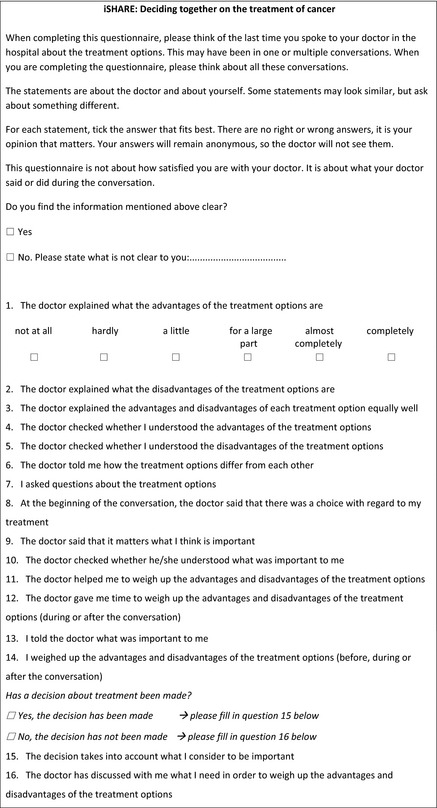

^†^This is an English translation of the original Dutch iSHAREpatient questionnaire. A translation agency translated the iSHAREpatient using a forward‐backward approach.

Box 3 **iSHAREphysician^†^**

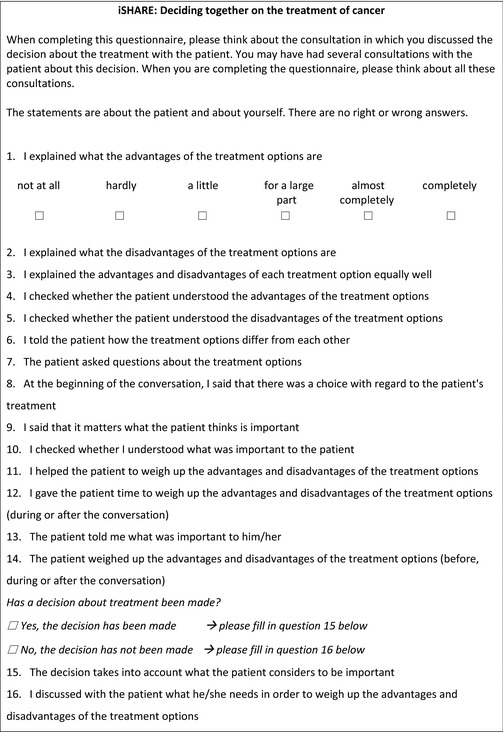

†This is an English translation of the original Dutch iSHAREphysician questionnaire. The translation is based on the translation of the iSHAREpatient.

The weighing of advantages and disadvantages of treatment options *during* and *outside* consultations is combined in one item, since patients can do either and do not need to do both. We recommend to assess the time at which patients have weighed treatment options separately, if researchers wish to explore this issue.

We assumed a formative measurement model, and therefore, the most appropriate scores to report on the iSHARE questionnaires are scores per dimension. Dimension scores can be calculated by averaging the scores on the relevant items (range scores, 0‐5). It may be useful to calculate a total score on the questionnaire, which then equals the sum of the scores on the dimensions (range total score, 0‐30). Higher scores per dimension and higher total scores indicate higher levels of SDM. A 0‐100 total score may be more intuitive, and we therefore recommend a linear transformation of the total score using the following formula: (score/30)*100.

## 4 DISCUSSION

In this study, we designed the iSHAREpatient and the iSHAREphysician questionnaires to assess SDM in oncology, based on a thorough development process. The iSHARE questionnaires contain the same items, formulated from the two different viewpoints. Both questionnaires assess patient as well as physician behaviours and aim to assess the SDM process during all consultations relevant to making the decision as well as during time outside of consultations. The iSHARE questionnaires may be used simultaneously or separately in future studies, depending on the research question. We decided that it would be most feasible for future studies if the two questionnaires would contain the smallest possible number of items. Throughout the development process, we therefore constantly prioritized domains and items, using the input provided by SDM experts, patients and physicians. Further, SDM measurement instruments from a patient viewpoint often seem to assess satisfaction rather than the extent to which SDM occurred.^18^ We made every effort to clarify to patients that the questionnaire is not about satisfaction, by making this explicit in the introduction of the questionnaire. The iSHARE questionnaires were developed for oncology. Yet, they are not formulated in ways that are specific to oncology and the questionnaires may thus prove useful in other settings as well. Use of the iSHARE questionnaires to assess SDM in non‐Dutch cancer settings and/or in other disease settings requires additional content validity testing.

The iSHARE questionnaires have some distinguishing features. First, the total score is not a function of who makes the final decision. This is consistent with our underlying SDM construct and reflects a finding from our earlier qualitative study. Specifically, in SDM in oncology it seems of minor importance *who* makes the final call, as long as the process was shared.^11^ Such an approach to SDM has been described by others. That is, patients were aware of and benefited from an SDM process, regardless of who* *they believed made the treatment decision.^31^ Second, the iSHARE questionnaires focus on an SDM process that can extend beyond a consultation. The iSHARE questionnaires therefore can be administered at various time points during the decision‐making process.

We started out with the assumption that the assessment of SDM should be based on a formative measurement model, as did the developers of the CollaboRATE^32^ and the OPTION‐5.^12^ Assuming a formative measurement model implicates the use of less regular methods to inform item reduction, one of which is rating the importance of items during field‐testing.^1^ In our study this method proved a feasible and valuable approach, but it would have been helpful to have specific, evidence‐based criteria to apply to the results when narrowing down the item pool. Measuring a construct based on a formative measurement model also implies that the calculation of a total score may not be appropriate, since the dimensions can be independent. Scores are therefore calculated per dimension. Clearly, a total score may sometimes be preferred because it can be a useful summary score. For the present questionnaires, we have no theoretical indication that one or more dimensions should be weighted differently from the others to calculate the total score.^1^, ^33^

Current measurement instruments assessing SDM from different viewpoints use the same items, formulated from different viewpoints, but agreement has nevertheless been found to be poor. We also used the same items for the iSHARE questionnaires, but let both patients’ and physicians’ views inform the SDM model which we used as input to our SDM construct. Further, both patients and physicians were involved in selecting the domains and items. With these questionnaires, we further ask participants about behaviour, and responses should therefore provide a view on what actually happened during decision‐making processes. We therefore expect that the iSHAREpatient and iSHAREphysician questionnaires will show at least a somewhat better agreement than has been found before.^14^, ^15^, ^19^ Nonetheless, interpretation of specific behaviours may still differ between patients and physicians, leading to different views on the extent to which SDM occurred.

We are currently undertaking a validation study to determine whether the iSHAREpatient and iSHAREphysician assess the construct as intended and assess SDM in similar ways from the two different viewpoints. Further assessment of psychometric properties of the questionnaires is necessary before recommending the use of the iSHARE questionnaires.

### 4.1 Study limitations

Although we used the original COSMIN checklist as a guideline throughout the development process,^28^ our findings should be considered in the light of two main limitations. First, physicians only assessed content validity on domain level and not on item level, for pragmatic reasons. Second, although we aimed to include patients representing a range of different education levels, most included patients were highly educated, resulting in potential biases towards domains and items that may be less important to or less comprehensible for other patients.

## 5 CONCLUSION

This study provides a patient and a physician questionnaire to assess SDM in oncology, based on a clearly defined construct and a thorough development process. The iSHARE questionnaires are short, assess both patient and physician behaviours, focus on the SDM process during all consultations relevant to making the decision, on the SDM process occurring outside consultations, and may be administered before or after the final decision has been made. Results obtained by using these questionnaires provide starting points to support the SDM process in ways tailored to actual behaviours and to both participants in the process.

## CONFLICT OF INTEREST

None.

## ETHICAL STATEMENT

The Medical Ethical Committee of the Leiden University Medical Center (LUMC) approved the study, which was conducted according to the Dutch Medical Research Involving Human Subjects Act.

## Data Availability

The data that support the findings of this study are available from the corresponding author upon reasonable request.

## APPENDIX 1

The construct of SDM in oncology; selection of clustered domains during the development process. Clustering is indicated by shading.

**Table:**

First selection informed by a model of SDM in oncology^11^ and a review of SDM models^5^(§3.2)	Selection after receiving feedback from SDM experts (§3.2)	Selection after content validity testing in cancer patients (§3.3)	Selection after content validity testing in physicians and field‐testing in cancer patients (§3.4)	Final selection after cognitive interviews in cancer patients and physicians (§3.6, Box 1)
The physician establishes (creates or checks) choice awareness	The physician establishes (creates or checks) choice awareness	The physician establishes (creates or checks) choice awareness	The physician establishes (creates or checks) choice awareness	The physician establishes (creates or checks) choice awareness
The physician expresses that patient’s opinion is important in process	The physician expresses that patient’s opinion is important in process	The physician expresses that patient’s opinion is important in process	The physician expresses that patient’s opinion is important in process	The physician expresses that patient’s opinion is important in process
The physician invites the patient to share decisions				
The physician decides on the agenda for the consultation				
	The physicians lists the treatment options	The physicians lists the treatment options	The physicians lists the treatment options^a^	
The physician provides information on the benefits/risks of the treatment options	The physician provides information on the benefits/risks of the treatment options	The physician provides information on the benefits/risks of the treatment options	The physician provides information on the benefits/risks of the treatment options	The physician provides information on the benefits/risks of the treatment options
The physician informs the patient that more than one option is medically acceptable				
The physician provides balanced information	The physician provides balanced information	The physician provides balanced information	The physician provides balanced information	The physician provides balanced information
	The physician checks patient’s understanding	The physician checks patient’s understanding	The physician checks patient’s understanding	The physician checks patient’s understanding
	The patient asks about (other) management options	The patient asks about (other) management options		
	The patient asks for clarification	The patient asks for clarification	The patient asks for clarification	The patient asks for clarification
The physician learns about the patient’s values, goals of care, concerns and/or preferences in the context of the treatment options	The physician learns about the patient’s values, goals of care, concerns and/or preferences in the context of the treatment options	The physician learns about the patient’s values, goals of care, concerns and/or preferences in the context of the treatment options	The physician learns about the patient’s values, goals of care, concerns and/or preferences in the context of the treatment options^a^	
	The physician checks own understanding of patient’s values, goals of care, concerns and/or preferences in the context of the treatment options	The physician checks own understanding of patient’s values, goals of care, concerns and/or preferences in the context of the treatment options		The physician checks own understanding of patient’s values, goals of care, concerns and/or preferences in the context of the treatment options
The patient expresses values, feelings, concerns, thoughts and preferences in the context of the treatment options	The patient expresses values, feelings, concerns, thoughts and preferences in the context of the treatment options	The patient expresses values, feelings, concerns, thoughts and preferences in the context of the treatment options	The patient expresses values, feelings, concerns, thoughts and preferences in the context of the treatment options	The patient expresses values, feelings, concerns, thoughts and preferences in the context of the treatment options
Physician supports with considering options	The physician supports the patient in deliberation	The physician supports the patient in deliberation	The physician supports the patient in deliberation	The physician supports the patient in deliberation
Physician deliberates				
The patient considers what is most important to him/her in the context of the treatment options	The patient considers what is most important to him/her in the context of the treatment options	The patient considers what is most important to him/her in the context of the treatment options, during the consultation	The patient considers what is most important to him/her in the context of the treatment options^a^	The patient considers what is most important to him/her in the context of the treatment options
		The patient considers what is most important to him/her in the context of the treatment options, before or after the consultation		
The physician gives a treatment recommendation that is a function of patient’s preferences	The physician gives a treatment recommendation that is a function of patient’s preferences	The physician gives a treatment recommendation that is a function of patient’s preferences		
The patient offers his/her opinion regarding the treatment options	The patient offers his/her opinion regarding the treatment options	The patient offers his/her opinion regarding the treatment options		
Make or explicitly postpone decision that is based on patient’s preferences/values/goals	Make or explicitly postpone decision that is based on patient’s preferences/values/goals	Make or explicitly postpone decision that is based on patient’s preferences/values/goals	Make or explicitly postpone decision that is based on patient’s preferences/values/goals	Make or explicitly postpone decision that is based on patient’s preferences/values/goals
	The physician assesses what the patient needs to make a decision	The physician assesses what the patient needs to make a decision	The physician assesses what the patient needs to make a decision	The physician assesses what the patient needs to make a decision
		The physician gives the patient room to contribute to SDM	The physician gives the patient room to contribute to SDM	The physician gives the patient room to contribute to SDM

^a^These domains were not selected for the draft physician questionnaire that was used as input to the cognitive interviews.
